# Effect of NiO Addition on the Sintering and Electrochemical Properties of BaCe_0.55_Zr_0.35_Y_0.1_O_3-δ_ Proton-Conducting Ceramic Electrolyte

**DOI:** 10.3390/membranes14030061

**Published:** 2024-02-27

**Authors:** Chengxin Peng, Bingxiang Zhao, Xie Meng, Xiaofeng Ye, Ting Luo, Xianshuang Xin, Zhaoyin Wen

**Affiliations:** 1 School of Materials and Chemistry, University of Shanghai for Science and Technology, Shanghai 200093, China; cxpeng@usst.edu.cn (C.P.); bxzhaobx@163.com (B.Z.); 2 The State Key Laboratory of High Performance Ceramics, Superfine Microstructure Shanghai Institute of Ceramics, Chinese Academy of Sciences, Shanghai 200050, China; mengxie@mail.sic.ac.cn (X.M.); yexf@mail.sic.ac.cn (X.Y.); luoting@mail.sic.ac.cn (T.L.); 3 Center of Materials Science and Optoelectronics Engineering, University of Chinese Academy of Sciences, Beijing 100049, China

**Keywords:** proton ceramic fuel cells, BaCe_0.55_Zr_0.35_Y_0.1_O_3-δ_ electrolyte, NiO sintering additive, electrochemical performance, sintering activity

## Abstract

Proton ceramic fuel cells offer numerous advantages compared with conventional fuel cells. However, the practical implementation of these cells is hindered by the poor sintering activity of the electrolyte. Despite extensive research efforts to improve the sintering activity of BCZY, the systematic exploration of the utilization of NiO as a sintering additive remains insufficient. In this study, we developed a novel BaCe_0.55_Zr_0.35_Y_0.1_O_3-δ_ (BCZY) electrolyte and systematically investigated the impact of adding different amounts of NiO on the sintering activity and electrochemical performance of BCZY. XRD results demonstrate that pure-phase BCZY can be obtained by sintering the material synthesized via solid-state reaction at 1400 °C for 10 h. SEM analysis revealed that the addition of NiO has positive effects on the densification and grain growth of BCZY, while significantly reducing the sintering temperature required for densification. Nearly fully densified BCZY ceramics can be obtained by adding 0.5 wt.% NiO and annealing at 1350 °C for 5 h. The addition of NiO exhibits positive effects on the densification and grain growth of BCZY, significantly reducing the sintering temperature required for densification. An anode-supported full cell using BCZY with 0.5 wt.% NiO as the electrolyte reveals a maximum power density of 690 mW cm^−2^ and an ohmic resistance of 0.189 Ω cm^2^ at 650 °C. Within 100 h of long-term testing, the recorded current density remained relatively stable, demonstrating excellent electrochemical performance.

## 1. Introduction

Solid oxide fuel cells (SOFCs) are highly promising power generation devices known for their high efficiency and low environmental impact. Currently, SOFCs are in the developmental phase for low-temperature operation (400–750 °C) [1]. The solid electrolyte of an SOFC is a critical component, as it determines the working temperature range. In contrast with traditional oxide-ion-conducting electrolytes, like 8 mol% Y_2_O_3_-stabilized ZrO_2_ (8YSZ) with a working temperature range of 750–1000 °C, proton-conducting electrolytes exhibit higher ionic conductivity and lower proton activation energy at lower temperatures [2,3]. SOFCs composed of proton-conducting electrolytes are commonly referred to as proton ceramic fuel cells (PCFCs), which are better suited for practical applications at lower temperatures [4,5,6]. PCFCs offer numerous advantages, including lower operating temperatures, faster start-up times, improved thermal cycle stability, and greater fuel flexibility [7,8]. These attributes make PCFCs an ideal choice for energy conversion at lower temperatures and hold the potential for significant roles in future practical applications [9].

BaCeO_3_/BaZrO_3_ oxides and hexagonal perovskites have been widely studied as proton conductors [10,11,12,13]. BaCeO_3_-based oxides exhibit excellent proton conductivity at intermediate temperatures, with a low proton conduction activation energy (0.4–0.6 eV) compared with the activation energy for oxygen ion transport [14,15]. However, at the actual PCFC operating temperature, BaCeO_3_-based oxides are unstable in the presence of acidic gases such as CO_2_ and/or steam [15]. In contrast, BaZrO_3_-based oxides demonstrate high chemical stability but low ionic conductivity [16,17]. Hence, researchers have proposed solid solutions between BaCeO_3_ and BaZrO_3_, in order to strike a balance between conductivity and chemical stability. In recent years, it has been confirmed that solid solution materials composed of BaCeO_3_ and BaZrO_3_ can achieve a favorable balance between corrosion resistance and conductivity [18,19]. For instance, the proton conductor BaZ_r0.1_Ce_0.7_Y_0.2_O_3-δ_ has been demonstrated to possess sufficient chemical stability under mild conditions. Furthermore, this BCZY material has been discovered to exhibit higher proton conductivity than the majority of oxygen-ion-conducting oxides at intermediate temperatures [20].

However, achieving the desired density in these solid solutions often requires higher sintering temperatures (>1600 °C) and longer dwell times [21]. Additionally, the fabrication of dense BCZY electrolyte membranes for PCFC typically involves a high-temperature sintering process. The high-temperature sintering leads to some critical problems, including severe thermal treatment conditions and challenges in the selection of compatible materials [22]. More importantly, high-temperature conditions can readily cause the volatilization and loss of Ba from the A site of the perovskite structure, resulting in alterations to the chemical composition of the electrolyte, which decreases its conductivity and stability [23]. Therefore, lowering the sintering temperature of the electrolyte is crucial for the practical application of PCFCs to advance.

In recent years, various transition metal oxides, such as ZnO, CoO, CuO, PdO, and NiO, have been widely used as additives to enhance the performance of BCZY electrolytes, particularly their sintering activity. The addition of ZnO can effectively promote the sintering of BaCeO_3_ and BaZrO_3_-based composites through a reaction between ZnO and BaO [24,25]. However, reports by Balibo and Haile suggest that the addition of ZnO may slightly decrease the conductivity of BZY [26]. Research by Ricote and Bonanos has demonstrated that doping with 1–2 mol% low concentrations of cobalt and nickel can significantly improve the sintering activity of BaZr_0.9_Y0.1O_3_ (BZY), confirming the beneficial effects of Co or Ni on the sintering of BZY while also introducing p-type conduction is important [27]. Similarly, Tan et al. [28] have discovered that the addition of 2.5 wt.% Co_2_O_3_ to the BCY perovskite proton conductor results in the formation of a well-defined BaCeO_3_ perovskite phase at 1250 °C, with tightly packed bulk grains and excellent mechanical properties. Tong et al. have suggested that CuO and PdO dopants can enhance total conductivity [10]. Despite extensive research on the sintering mechanisms of transition metal oxides as sintering aids for proton-conductive electrolyte materials, there is still limited exploration of the impact of NiO on the sintering activity and electrochemical performance of BCZY electrolytes. According to research by Liu et al. [29], BCZY and NiO form a BaY_2_NiO_5_ phase at temperatures above 1000 °C, and begin to form sintered regions at grain boundaries, promoting grain growth and densification. To further advance research and applications in this field, it is necessary to elucidate the influence of NiO sintering aids on the sinterability and conductivity of BCZY proton-conductive ceramic electrolytes.

In this study, a BaCe_0.55_Zr_0.35_Y_0.1_O_3-δ_ (BCZY) electrolyte was developed, and the impact of adding different amounts of NiO as a sintering aid on the phase structure and sintering activity of BCZY was systematically investigated. Additionally, proton ceramic fuel cells were fabricated and tested using BCZY electrolyte with varying amounts of NiO. The electrochemical performance, electrochemical impedance spectroscopy (EIS), and stability of the fuel cell were thoroughly investigated under real operating conditions.

## 2. Experimental Section

### 2.1. Sample Preparation

The BaCe_0.55_Zr_0.35_Y_0.1_O_3-δ_ (BCZY) electrolyte powder was synthesized via a solid-state reaction. BaCO_3_ (National Pharmaceutical, 99.95%), CeO_2_ (Aladdin, 99.99%), ZrO_2_ (Aladdin, 99.99%), Y_2_O_3_ (Aladdin, 99.99%), and NiO (National Pharmaceutical, 99.99%) were used as the raw materials. The initial powder mixture was manually blended, placed in a ball mill tank, and then ball milled in a planetary ball mill using ethanol as the solvent and zirconia balls as the medium for 24 h to obtain the mixed powder. After ball milling, the mixed powder was dried in an oven at 80 °C. To determine the optimal temperature for stable perovskite phase formation, the BCZY electrolyte powder was heated in the temperature range of 900–1400 °C for 10 h. Subsequently, BCZY powders were prepared by sintering at 1150 °C for 10 h. For comparative purposes, 0.0 wt.%, 0.5 wt.%, 1.0 wt.%, 1.5 wt.%, and 2.0 wt.% of NiO were added to the BCZY electrolyte powder, followed by 2 h of ball milling. The mixtures were then dried in an oven and labeled as BCZY-0, BCZY-0.5, BCZY-1.0, BCZY-1.5, and BCZY-2.0, respectively. Subsequently, a certain amount of PVA binder was added to the powders, and the powders were pressed into pellets with a radius of 20 mm and at a pressure of 100 MPa using a uniaxial press. The relative densities and conductivities of the samples were tested after sintering at 1350 °C for 10 h. Shrinkage was measured by testing the compacted electrolyte powder strips. It is important to note that all samples were buried and sintered using BCZY electrolyte powder during the process.

### 2.2. Single Cells Fabrication

Anode-supported single cells were fabricated using uniaxial pressing and screen-printing techniques, and all of the single cells consisted of the following five layers: anode support, anode functional layer, electrolyte, cathode functional layer, and cathode current collector. Firstly, the anode support was prepared by mechanically mixing NiO, BCZY, and graphite with 6:4:1 for 2 h of ball milling. The anode functional layer was prepared by mechanically mixing 60 wt.% NiO and 40 wt.% BCZY powder for 2 h of ball milling. The two layers were then uniaxially pressed under a pressure of 100 MPa for 60 s to form an anode substrate. Subsequently, the electrolyte slurry with different amounts of NiO sintering aid (BCZY-0, BCZY-0.5, BCZY-1.0, BCZY-1.5, BCZY-2.0) was screen-printed on one side of the anode functional layer. The half-cell was then sintered at 1350 °C for 5 h. Lastly, the LSCF–BCZY (7:3, by mass) cathode functional layer and the cathode current collector (LSCF) were screen printed successively on the electrolyte surface, and the full cell was sintered at 900 °C for 3 h. The effective area of the cathode was 0.5 cm^2^.

### 2.3. Material Characterization

The phase compositions of the synthesized materials were determined through X-ray diffraction analysis using a Rigaku SmartLab SE instrument (XRD, UltimaIV, Rigaku, Tokyo, Japan). Using Jade6 software, the corresponding lattice volume, and unit cell constants, as well as the corresponding proportions of each phase, were calculated through refinement technology. SEM analysis (ZEISS) was performed to examine the morphology of the electrolyte surface and the cross-section of the PCFC. To assess the impact of different NiO concentrations on the sintering performance of BCZY, a high-temperature dilatometer (DIL402PC, NETZSCH) was used to measure the linear shrinkage of BCZY-xNiO (x = 0, 0.5, 1.0, 1.5, 2.0) in air from room temperature to 1400 °C, with a heating rate of 5 °C min^−1^. The linear shrinkage (%) of sintered ceramic samples was determined using Equation (1):

(1) $$Shrinkage\;\%=\frac{∆L}{L_{0}}\times 100$$

The impact of different NiO addition levels on the relative density of BCZY ceramic samples was also assessed using Archimedes’ displacement method. The relative density was calculated using Equation (2):

(2) $$P=1-\frac{\left(m_{2}-m_{1}\right)}{\left(m_{2}-m_{3}\right)}\times 100\%$$

where *m*_1_ represents the dry weight of the ceramic sample, *m*_2_ represents the wet weight of the ceramic sample after soaking in distilled water for 24 h, and *m*_3_ represents the weight of the ceramic sample under the buoyant force of distilled water.

### 2.4. Electrical Property Measurement

The AC impedance technique was applied to the characterization of electric conductivity by the Zahher electrochemical workstations.

The electrical conductivity of the ceramic pellets was calculated using Equation (3):

(3) $$\sigma =\frac{D}{RS}$$

where σ = conductivity, D = thickness of sample, R = resistance, and S = surface area. 

The activation energy was calculated using the Arrhenius plot with Equation (4):

(4) $$\sigma =\frac{A}{T}\exp\left(\frac{E_{a}}{K_{B}T}\right)$$

where σ = conductivity, A = pre-exponential factor, $E_{a}$ = activation energy, $K_{B}$ = Boltzmann constant, and T = absolute temperature.

The single cell was tested in the temperature range of 600–700 °C, using humidified H_2_ (3% H_2_O, 40 mL min^−1^) and ambient air in a static environment as the fuel and oxidant, respectively. The durability of the cell was tested at a fixed voltage of 0.7 V at 650 °C.

## 3. Results and Discussion

### 3.1. X-ray Diffraction Analysis

**Figure 1:**
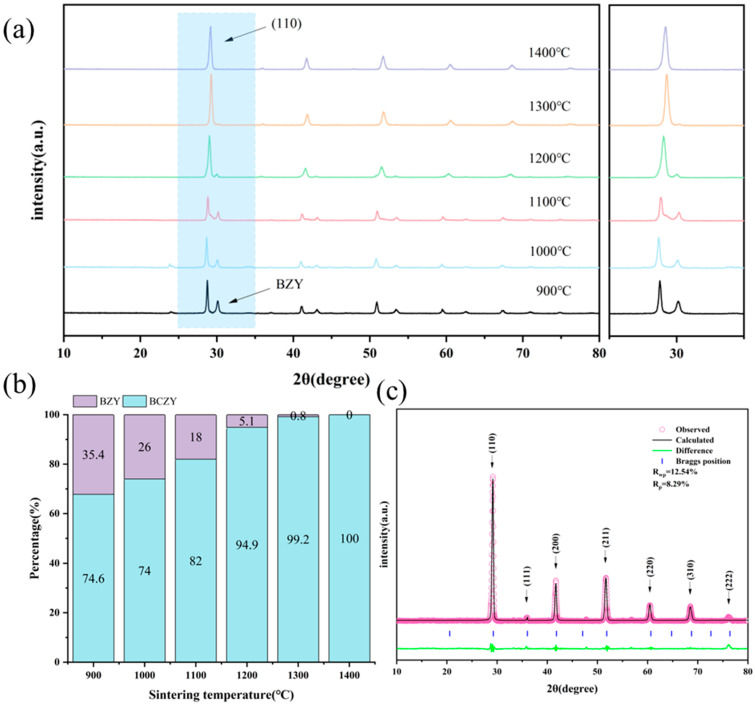
X-ray diffraction analysis of BCZY powder sintered at 900–1400℃ for 10h. (**a**) XRD patterns of BCZY electrolyte powder sintered at 900–1400 °C for 10 h, (**b**) phase composition and content of BCZY electrolyte powder sintered at 900–1400 °C for 10 h, and (**c**) refined XRD pattern of pure-phase BCZY obtained after sintering at 1400 °C for 10 h.

XRD phase analysis of the synthesized materials is presented in Figure 1. Figure 1a shows that BCZY underwent phase transformation and formed a perovskite phase after being sintered at 1000 °C for 10 h. However, the perovskite phase was not pure and was accompanied by a significant proportion of the BZY phase. Given that BCZY requires a higher sintering temperature to achieve a fully formed perovskite phase, the presence of BZY in the sample can be considered as an “intermediate transition phase” during the sintering process. Based on the refined calculation of the content ratio of each phase depicted in Figure 1b, it is evident that the BZY content gradually diminishes with increasing sintering temperature, ranging from 35.4 wt.% at 900 °C to 0.8 wt.% at 1300 °C. Given this trend, it can be concluded that a pure-phase BCZY perovskite structure can only be achieved by sintering at 1300 °C and above for a duration of 10 h.

Additionally, when examining the enlarged peak in the range of 25–35°, it is observed that, as the sintering temperature increased, the (110) crystal plane continuously shifted to higher angles. This phenomenon can be primarily attributed to the gradual substitution of Zr into the BCZY lattice during the sintering process. From an ionic radius perspective, Zr^4+^ has a smaller ionic radius of 0.72 Å compared with Ce^4+^ (0.87 Å) and Y^3+^ (0.90 Å) at the same B-site. As a result, the gradual incorporation of Zr^4+^ into the BCZY lattice, replacing Ce^4+^ and Y^3+^, causes a higher angle shift in the BCZY diffraction peak. Refined XRD results confirm that the pure-phase BCZY formed at 1400 °C has a cubic perovskite structure with a space group of Pm-3m and a lattice constant of 4.327 Å.

**Figure 2:**
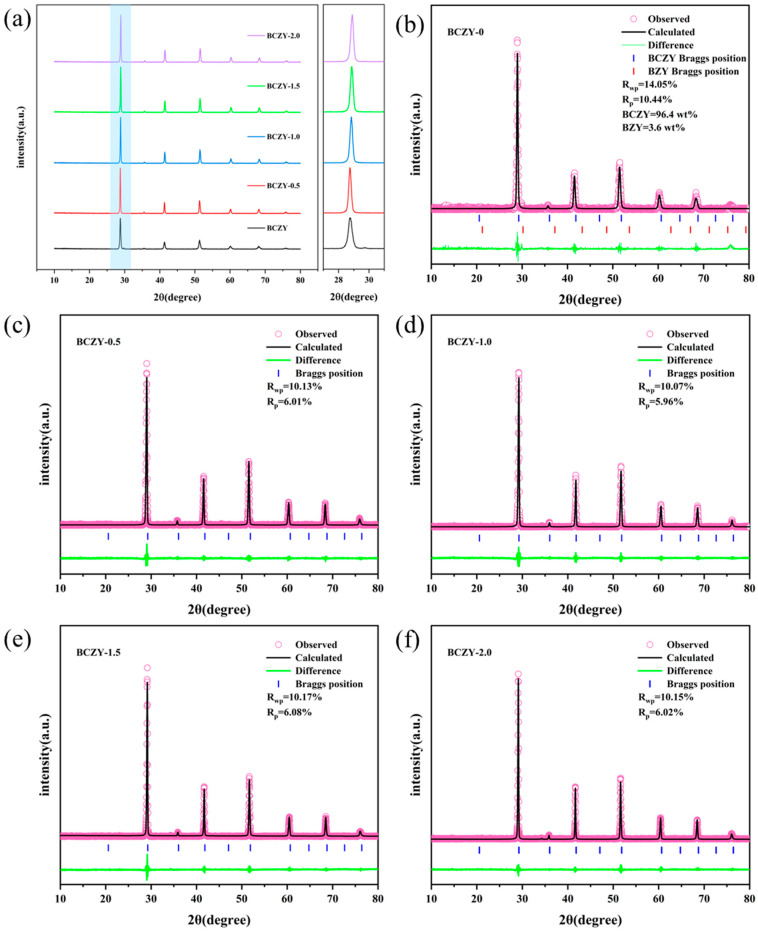
(**a**) XRD diffraction patterns of BCZY electrolyte pellets sintered at 1350 °C for 5 h with varying amounts of NiO and an enlarged region in the range of 27–31°. Refinement results for the XRD patterns of samples: (**b**) BCZY-0, (**c**) BCZY-0.5, (**d**) BCZY-1.0, (**e**) BCZY-1.5, and (**f**) BCZY-2.0.

Figure 2a illustrates that the addition of NiO causes the (110) crystal plane to shift at a larger angle, suggesting that Ni^2+^ (0.69 Å), with a smaller ionic radius, could potentially replace some B-site elements in the BCZY perovskite structure. Additionally, Figure 2b–f demonstrate that the lattice parameters of BCZY, determined using JADE software for refinement, decrease progressively with the inclusion of NiO. The impact of NiO addition on the crystal structure of BCZY is evident in the reduction of its lattice parameters. The lattice parameters range from 4.3736 Å to 4.3692 Å with the incorporation of a small quantity of NiO (0.5 wt.%). Table 1 presents a detailed overview of the changes in lattice parameters of BCZY upon the addition of NiO. It is important to highlight that Ni, as a transition metal, has the potential to occupy the B site in the ABO_3_ perovskite structure, thus replacing certain ions in the original crystal’s B position. The ionic radius of Ni^2+^ (0.69 Å) is smaller than the Zr^4+^ (0.72 Å), Ce^4+^ (0.87 Å), and Y^3+^ (0.90 Å) located at the B position. Therefore, the decrease in the lattice parameters of Ni-doped BCZY indicates that Ni has replaced part of Zr, Ce or Y.

### 3.2. Sintering Behavior

**Figure 3:**
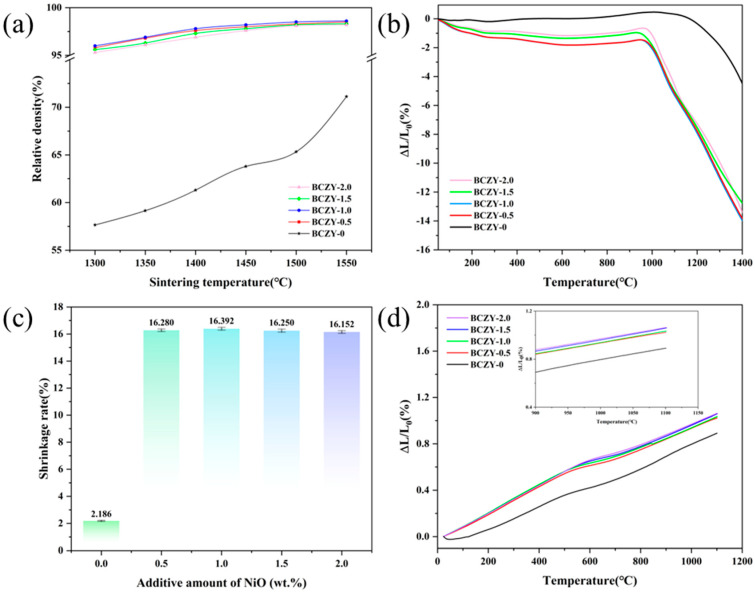
Sintering behavior test of BCZY electrolyte pellets with different amounts of NiO added. (**a**) Relative density of BCZY electrolyte pellets with different amounts of NiO sintered at 1300–1550 °C for 5 h, (**b**) shrinkage curves of BCZY and BCZY with different amounts of NiO, (**c**) shrinkage rate of electrolyte pellets after sintering at 1350 °C for 5 h, and (**d**) thermal expansion curves of BCZY and BCZY with different NiO addition amounts.

The samples underwent sintering behavior testing, and the data obtained provided valuable insights into the sintering behavior and densification of the material under specified sintering conditions. By analyzing the shrinkage results, it is possible to assess the effectiveness of the sintering process and evaluate the achieved level of compaction in the BCZY sample. Figure 3a illustrates the variation in relative density of BCZY with different amounts of NiO, which were sintered and held at temperatures ranging from 1300 °C to 1550 °C for 5 h. It is observed that BCZY exhibits low relative density within the entire range of sintering temperatures. However, the addition of even a small amount of NiO has a significant impact on increasing the relative density of BCZY. After sintering at 1300 °C for 5 h, BCZY-0 has a relative density of only 57.65%, while BCZY-0.5 and BCZY-1.0 reach 95.93% and 96.12% in relative density, respectively. BCZY-1.5 and BCZY-2.0 show a slight decrease in relative density but still achieve 95.8% and 95.6%. The results obtained in this study align with the findings reported by Liu et al. [29], highlighting a similarity in the observed outcomes. It is hypothesized that the presence of impurity phases, potentially caused by an excessive amount of NiO addition, can have a detrimental effect on the sintering activity of BCZY.

As the sintering temperature continues to increase, the relative density of BCZY-0 steadily improves, but the relative density is still only 71.12% at 1550 °C. Despite the significance of sintering temperature in densification, the poor sintering activity of BCZY hinders its ability to achieve high density, even at a temperature as high as 1550 °C. In contrast, BCZY-0.5, BCZY-1.0, BCZY-1.5, and BCZY-2.0 each show a different behavior. These compositions reach their highest relative density at around 1450 °C, and there is little variation in relative density as the temperature increases further. The relative density for these compositions remains relatively high, at approximately 97%-98%, indicating that they have better sintering activity compared with BCZY-0.

Furthermore, as shown in Figure 3b, the sintering behavior of BCZY can be characterized as a function of temperature by using the thermal dilatometer, tested in the range of 50–1400 °C with a heating rate of 5 °C/min. It can be observed that BCZY-0 gradually starts to shrink at around 1100 °C and reaches a total shrinkage of 8.7% at 1400 °C. Adding NiO sintering aids to BCZY can indeed reduce the starting shrinkage temperature to 1000 °C, allowing for a lower sintering temperature, which can be advantageous in terms of energy consumption and processing time. Additionally, the rapid shrinkage observed in the temperature range of 1000 °C to 1400 °C indicates the effectiveness of the NiO sintering aid in promoting densification and achieving a high degree of sintered compaction. This suggests that the presence of NiO aids in the sintering process and promotes the densification of the BCZY material at lower temperatures. The shrinkage curves indicate that BCZY-0.5, BCZY-1.0, BCZY-1.5, and BCZY-2.0 exhibit shrinkage rates of 13.99%, 14.12%, 12.90%, and 13.64%, respectively, at the same temperature interval. Results demonstrate that the inclusion of a NiO sintering additive can effectively enhance the sintering performance of BCZY.

The results of the shrinkage test conducted on the sample at a sintering temperature of 1350 °C for 5 h are shown in Figure 3c and involved measurement of the dimensional changes in the sample using a micrometer. BCZY-0, BCZY-0.5, BCZY-1.0, BCZY-1.5, and BCZY-2.0 exhibit shrinkage rates of 2.19%, 16.23%, 16.35%, 16.20%, and 16.11%, respectively. The addition of a small amount of 1 wt.% NiO sintering additive yields the highest shrinkage rate, while further increasing the NiO content leads to a slight reduction in the shrinkage rate, corresponding with the relative density results.

Currently, most PCFCs use cobalt-rich cathodes, such as LSCF [30], BSCF [5,31], BCFZY [32,33], and PNC [34], etc. Thermal compatibility is a critical issue with these cobalt-rich cathodes, which typically exhibit high thermal expansion coefficient (TEC) values within the operating temperature range. The high TEC of these cobalt-based cathodes is primarily due to the spin state transition of cobalt and oxygen loss at high temperatures. A mismatch in TEC between the electrolyte and cathode can cause delamination at the cathode–electrolyte interface or cracking in the electrolyte. Therefore, the thermal expansion of both the electrolyte and cathode needs to be taken into account to prevent these issues. As depicted in Figure 3d, negligible differences exist in the thermal expansion coefficients of the various samples. The thermal expansion coefficient of BCZY, ranging from room temperature to 1100 °C, exhibits a minor increase corresponding to the NiO content increment. Among all of the tested specimens, BCZY possesses the lowest thermal expansion coefficient, recorded at 8.33 × 10^−6^ K^−1^. Conversely, BCZY-2.0 demonstrated the highest thermal expansion coefficient, measured at 9.91 × 10^−6^ K^−1^. This value aligns closely with the reported thermal expansion coefficient of cobalt-rich cathodes (12–16 × 10^−6^ K^−1^) [35], suggesting its potential compatibility with the cathode in real-world operational conditions.

### 3.3. Surface Morphology

**Figure 4:**
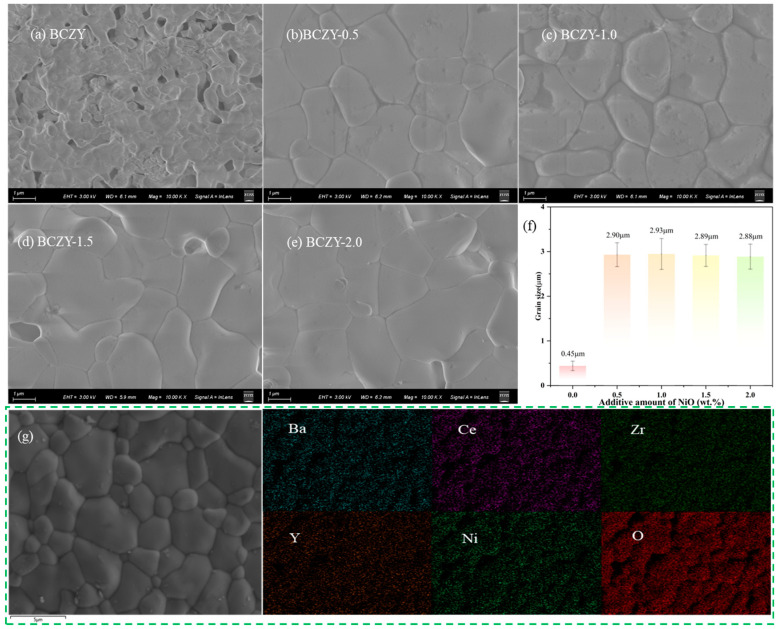
SEM images of BCZY electrolyte pellets with different amounts of NiO added after sintering at 1350 °C for 5 h. (**a**) BCZY-0, (**b**) BCZY-0.5, (**c**) BCZY-1.0, (**d**) BCZY-1.5, (**e**) BCZY-2.0, (**f**) statistical diagram of grain size, and (**g**) SEM–EDS mapping results for BCZY-1.0.

The SEM images in Figure 4a–e illustrate the ceramic electrolyte surface morphology after sintering at 1350 °C for 5 h. BCZY-0 exhibits a porous structure after sintering, while all BCZY samples with added NiO sintering aids appear nearly completely densified. The grain size significantly increases, indicating that NiO sintering aids effectively lower the sintering temperature and enhance densification. Figure 4f illustrates the variation in the average particle size for different compositions of BCZY–NiO. BCZY-0 exhibits the smallest average particle size at 0.45 μm. However, as the NiO content increases, the particle size increases significantly for BCZY-0.5, reaching 2.90 μm. The maximum particle size is observed for BCZY-1.0, with an average particle size of 2.93 μm. As the NiO content further increases, the particle size slightly decreases for BCZY-1.5 and BCZY-2.0. The improved sintering performance of added BCZY is likely due to the formation of a low melting point phase when NiO combines with BCZY [29,36]. This low melting point phase forms a liquid phase during sintering, promoting grain rearrangement and material transport. Furthermore, the sintering process is influenced by the difference in ionic radius between Ni^2+^ and Ce^4+^. The smaller ionic radius of Ni^2+^ compared with Ce^4+^ leads to lattice distortions when Ni^2+^ is incorporated into the BCZY lattice, which in turn increases the mobility of grain boundaries. This charge imbalance caused by the substitution of Ni^2+^ for Ce^4+^ at the B site also results in the creation of oxygen vacancies, which further affects grain growth and enhances grain boundary mobility.

Before conducting the conductivity testing, the elemental distribution of the samples was analyzed using SEM–EDS, which allows for the identification and mapping of different elements present in the sample, providing valuable information about their distribution and composition. Figure 4g shows the SEM–EDS results of BCZY-1.0 after sintering at 1350 °C for 5 h. The elemental analysis of BCZY-1.0 pellets sintered at 1350 °C revealed a relatively uniform distribution of elements. This is noteworthy considering the lower sintering temperature and holding time utilized in the study. Contrary to the findings reported by Wang et al. [21], no elemental segregation of Y or the appearance of yttrium-rich phases was observed in the tested samples. Moreover, the electrolyte pellet, which underwent buried firing during the sintering process, was effectively shielded. The test results verify the absence of barium volatilization after sintering was completed.

### 3.4. Conductivity

Based on the evaluation of the sintering behavior of various samples, it is observed that the addition of NiO to BCZY seems to have a limited effect, and even a small amount of addition can effectively enhance the sintering performance of BCZY. Consequently, electrochemical impedance spectroscopy (EIS) was employed to conduct conductivity tests on BCZY-0, BCZY-0.5, and BCZY-1.0. These samples were sintered at 1350 °C for 5 h, and the ionic conductivity was measured in dry air and humid hydrogen atmospheres (~3% H_2_O).

It was observed that the protonic conductivity in BCZY is closely related to operating temperature and environmental atmosphere. BCZY is widely recognized for its triple-conductivity, which involves the transport of protons, oxygen ions, and electron holes. The Grotthus “hopping” mechanism is widely accepted as the proton transport mechanism in BCZY [2,37]. In this mechanism, protons rotate and diffuse around oxygen atoms, creating hydroxide ions. These protons then jump from one lattice oxygen site to another, facilitating proton conduction. The mechanism entails protons attaching to an oxygen ion and moving together with it. The generated hydroxide ions continuously diffuse through the perovskite structure to complete proton migration.

**Figure 5:**
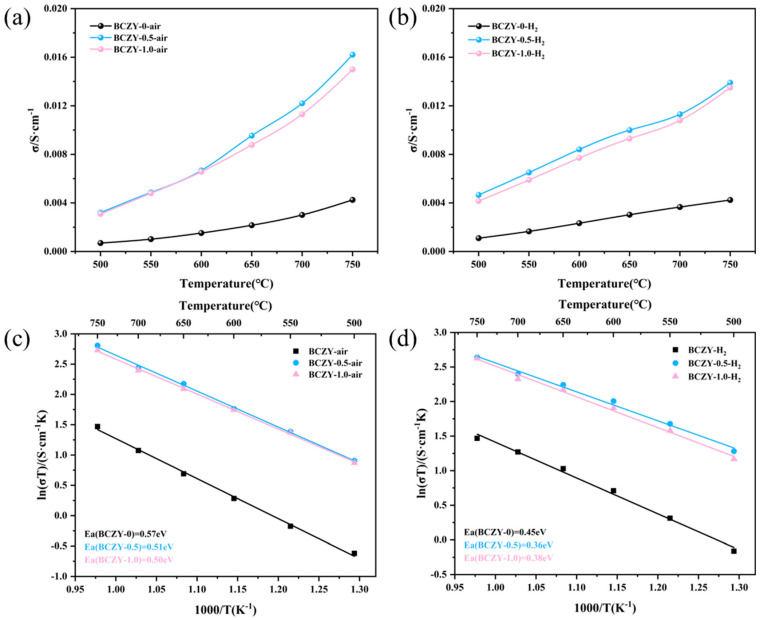
Electrical conductivity of BCZY, BCZY-0.5, and BCZY-1.0 in atmospheres of air (**a**) and wet hydrogen (**b**). Arrhenius curves in the air (**c**) and humidified hydrogen (**d**) atmospheres.

Figure 5a,b clearly demonstrate that the BCZY-0.5 electrolyte displays higher proton conductivity compared with other electrolytes. The specific values of conductivity are shown in Table S1 in the Supplementary Materials. Significantly, BCZY-1.0, with a higher content of NiO, displays lower proton conductivity than BCZY-0.5 in both atmospheres. In the case of ceramic materials, it is generally observed that higher bulk density correlates with greater electrical conductivity. This is because higher density results in a greater concentration of conductive pathways for the movement of electrons or ions within the material. Nevertheless, it is essential to acknowledge that conductivity can be influenced by various factors, including grain size, the number of grain boundaries, and the presence of impurities. Grain size is a factor that affects conductivity, as smaller grains can offer more grain boundaries, which can facilitate the movement of charge carriers. Furthermore, the presence of impurities or dopants can either improve or hinder conductivity, depending on their impact on the crystal structure and ion mobility. Therefore, although higher bulk density typically indicates higher electrical conductivity in ceramic materials, other factors, like grain size, grain boundaries, and impurities, can also impact conductivity to some degree.

In terms of sintering shrinkage, it is important to note that BCZY-1.0 exhibits higher relative density and shrinkage compared with BCZY-0.5. However, the increased NiO content in BCZY-1.0 may lead to a higher level of impurities. Adding 0.5 wt.% NiO as a sintering aid in BCZY electrolytes not only enhances the sintering process but also improves their electrochemical performance, which is then reflected in subsequent single-cell performance. Furthermore, it was observed that, in the low-temperature range of 500 °C to 650 °C, BCZY-0, BCZY-0.5, and BCZY-1.0 exhibited slightly higher proton conductivity in a humidified H_2_ atmosphere than in a dry air atmosphere. However, as the temperature increased, the proton conductivity in a humidified H_2_ atmosphere became lower than that in a dry air atmosphere. 

Figure 5c,d demonstrate that BCZY-0.5 displays an activation energy of 0.51 eV in a dry-air environment. This value decreases to 0.36 eV in a humidified H_2_ atmosphere. The reduced activation energy in a humidified H_2_ atmosphere, relative to the dry air environment, suggests that the conductivity in dry air is largely governed by the mixed conduction of oxygen ions and electron holes. In dry air, oxygen combines with oxygen vacancies to form oxygen ions and electron holes [38].

(5) $$V_{o}^{\cdot \cdot }+\frac{1}{2}O_{2}\to O_{o}^{\times }+2h^{\cdot }$$

In humidified hydrogen, proton conduction emerges as the primary mechanism, contrasting with the dry-air atmosphere where mixed conduction of oxygen ions and electron holes is dominant. This humid environment initiates a series of proton-related processes. The process begins with the ionization of water, leading to the formation of hydroxide ions (OH^−^) and protons (H^+^). These hydroxide ions fill the oxygen vacancies within the perovskite oxide structure. Concurrently, protons form chemical bonds with lattice oxygen, facilitating their entry into the perovskite structure and enabling their subsequent migration to adjacent oxide ions. The exothermic nature of the proton entry process from water into the perovskite oxide indicates that proton mobility decreases as temperature increases.

(6) $$H_{2}O+O_{o}^{\times }+V_{o}^{\cdot \cdot }\to 2{OH}_{o}^{\cdot }$$

Simultaneously, the ionization of hydrogen gas occurs. In humidified hydrogen, electronic conduction caused by electron holes is inhibited, facilitating proton conduction. Alongside this, the ionization of hydrogen gas also takes place. The humid environment suppresses the electronic conduction attributed to electron holes, thereby enhancing proton conduction.

(7) $$\frac{1}{2}H_{2}+O_{o}^{\times }\to {OH}_{o}^{\cdot }+e^{\prime }$$

Therefore, proton conductors typically demonstrate the ability to conduct protons, oxygen ions, and electrons. In an air atmosphere, the samples primarily function as mixed conductors of electronic holes and oxygen ions. However, in a humidified H_2_ atmosphere, they act as proton conductors. Additionally, the lower activation energy observed in humidified H_2_ indicates that proton conductors are more suitable for medium-to-low-temperature environments.

### 3.5. Electrochemical Performance of Single Cells

To evaluate the impact of NiO as a sintering aid in single-cell performance, fuel cells were tested under static air and humidified H_2_ (~3% H_2_O) conditions. It is worth noting that, while some studies have used sintering aids with BZCY materials, the majority of research has focused on investigating material properties such as sinterability and electrical conductivity. Few reports have delved into the performance of cells modified with sintering aids under actual fuel cell operating conditions. In contrast with previous research that has mainly emphasized material properties, this work investigates the feasibility of using BCZY electrolyte materials with added NiO as a PCFC electrolyte. All five cells were prepared using the same electrode materials and the same fabrication process, with the only variation being the electrolyte. Therefore, any performance differences among the different cells were solely attributed to the variations in the electrolyte. 

**Figure 6:**
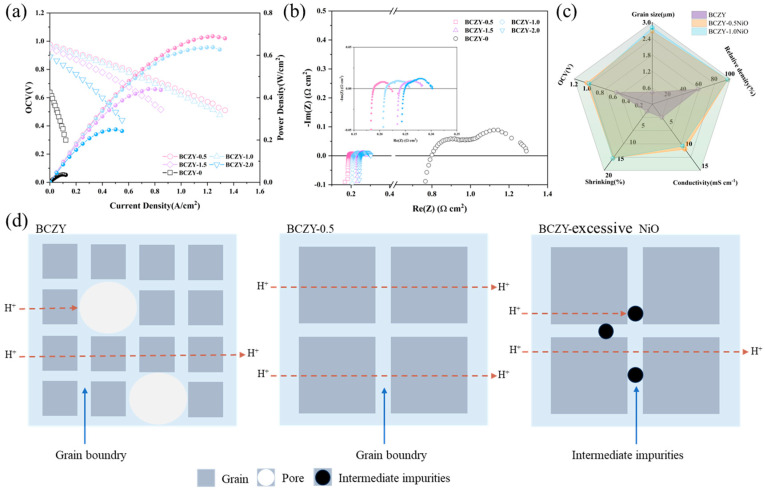
Single cell output performance diagram and proton transport diagram. (**a**) the single cell I–V and I–P diagrams of NiO–BCZY/BCZY-x (x = 0, 0.5, 1.0, 1.5, 2.0)/BCZY–LSCF with a cell structure; (**b**) EIS diagram; (**c**) performance comparison of BCZY, BCZY-0.5, and BCZY-1.0; (**d**) scheme for proton transportation in BCZY, BCZY-0.5 and BCZY-excessive NiO.

Figure 6a shows the I–V and I–P curves of single cells with BCZY-0, BCZY-0.5, BCZY-1.0, BCZY-1.5, and BCZY-2.0 as electrolytes at 700 ℃ (the test curves at 650 °C and 600 °C are shown in Figure S1 in the Supplementary Materials). The cell that employs BCZY-0.5 as the electrolyte demonstrates relatively excellent power output performance and attains a maximum power density of 690 mW cm^−2^ and an open circuit voltage (OCV) of 0.975 V when operating at 700 °C, nearing the theoretical electromotive force. The ohmic resistance of the cell is a mere 0.189 Ω cm^2^, which is primarily due to the dense electrolyte membrane, as shown in Figure 6b (the test curves of single cells with BCZY-0, BCZY-0.5, BCZY-1.0, BCZY-1.5, and BCZY-2.0 as electrolytes at 650 °C and 600 °C are shown in Figure S2). The inherently poor sintering activity of BCZY makes it challenging to densify. The open circuit voltage and maximum power density of BCZY-0 are only 0.638V and 38 mW cm^−2^, which are far lower than the power output performance of BCZY-0.5. It is important to note that the BCZY-1.0 electrolyte cell exhibits marginally lower OCV and maximum power density performance in comparison with BCZY-0.5. At 700 °C, its maximum power density is 638 mW cm^−2^, and the OCV is recorded at 0.963 V. In contrast, the BCZY-1.0 electrolyte demonstrates superior sintering activity, irrespective of shrinkage or relative density.

The schematic diagram in Figure 6d illustrates the smooth transfer of protons within BCZY-0.5 particles, while in BCZY-0 particles, protons may encounter more grain boundaries, thereby impacting proton transport. Furthermore, it is worth noting that the partial substitution of Ce^4+^ by Ni^2+^ in BCZY-0.5 can generate a greater number of oxygen vacancies compared with BCZY-0, as indicated by the following equation:

(8) $$NiO\overset{{BaCeO}_{3}}{\to }{Ni}_{Ce}^{\cdot \cdot }+V_{Ba}^{\cdot \cdot }+V_{o}^{*}+O_{o}$$

Therefore, the proton concentration in BCZY-0.5 is expected to be higher than that in BCZY-0, potentially leading to enhanced proton conduction. Considering the gradually increasing NiO content, it may introduce electronic conductivity within the electrolyte. Simultaneously, some of the unincorporated NiO or NiO formed as impurities such as BaY_2_NiO_5_, which, due to its reaction with BCZY, may become pinned at grain boundaries, thus acting as non-proton-conductive impurity phases that increase ohmic resistance and negatively impact cell performance [36]. With further increases in NiO content, maximum power density and OCV gradually decrease, suggesting that excess NiO reacts with more BCZY electrolytes, forming non-proton-conductive impurity phases, obstructing proton transport pathways, and compromising cell performance. The cell without added NiO exhibits the poorest electrochemical performance, which corresponds to its suboptimal sintering activity, resulting in small grain size and high porosity, and in turn leading to significant ohmic resistance. Meanwhile, during the co-firing process of the half-cell, the NiO in the anode part diffuses into the electrolyte layer at high sintering temperatures, contributing to the densification of the electrolyte [39]. This diffusion of Ni during co-firing confirms that increasing the NiO content in the electrolyte will lead to a reduction in the open-circuit voltage.

**Figure 7:**
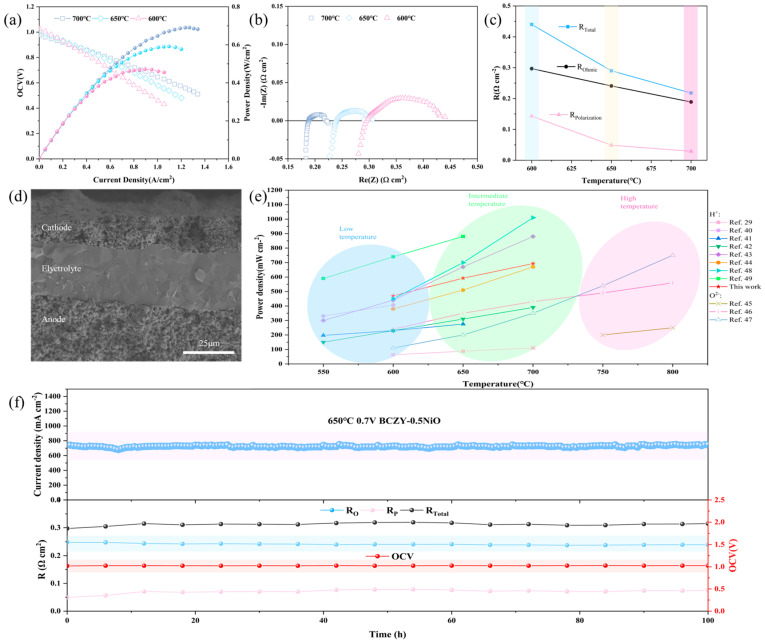
(**a**) I–V and I–P diagrams of the single cell with a structure of NiO-BCZY/BCZY-0.5/BCZY–LSCF at 600–700 °C, (**b**) EIS diagram, (**c**) the corresponding EIS of the complete single cell measured under open circuit conditions, (**d**) cross section view of the single cell after testing, (**e**) comparison of MPDs of SOFCs based on H^+^ and O^2−^ conductors [29,40,41,42,43,44,45,46,47,48,49], and (**f**) durability test of the cell at 650 °C and 0.7 V.

Figure 7a displays the I–V and I–P curves for the BCZY-0.5 full cell tested at 600–700 °C. At 700 °C, 650 °C, and 600 °C, the maximum power densities reach 690, 591, and 471 mW cm^−2^, with OCV of 0.975, 1.007, and 1.027V, respectively. The higher OCV is mainly due to the dense electrolyte membrane, which effectively prevents gas leakage. Figure 7b shows the impedance spectrum of the single cell with BCZY-0.5 as the electrolyte under OCV conditions. The ohmic resistance (*R_ohmic_*) values are measured to be 0.189, 0.241, and 0.297 Ω cm^2^ at 700, 650, and 600 °C, respectively. Figure 7d presents SEM micrographs of the tested single cell. The BCZY-0.5 electrolyte layer has a thickness of about 30 μm, adhering well to the anode and cathode layers. The electrolyte exhibits no visible pores or cracks, ensuring effective gas separation between the anode and cathode. As depicted in Figure 7c, the ohmic resistance constitutes the major portion of the total resistance, possibly caused by the thicker electrolyte membrane. With decreasing temperature, the significant fluctuations in total resistance (*R_total_*) primarily arise from the larger variations in polarization resistance (*R_polarization_*).

The durability test was performed on an anode-supported single cell operating at an open-circuit voltage of 0.7 V using humidified H_2_ fuel as given in Figure 7f. During the 100 h of durability testing, the current density remained at a relatively high level of 700–740 mA cm^−2^, and the recorded results show a stable trend overall. The open circuit voltage also demonstrated relatively stable performance. In addition, the OCV of the single cell operating under open circuit conditions was stable above 1 V, indicating that the electrolyte membrane maintained its dense structure throughout the test. This effectively prevented air leakage that could have been caused by a less dense electrolyte membrane. The surface of the electrolyte membrane showed a uniform distribution of elements without any element segregation after testing (Figure S3), indicating the stability of the electrolyte during long-term operation.

Furthermore, the ohmic resistance remained relatively constant during the extended testing period, essentially maintaining a value of approximately 0.240 Ω cm^2^. In contrast, the polarization resistance showed significant variation during the initial stages of cell operation, with a continuous increase over time, leading to overall impedance fluctuations. However, the polarization resistance stabilized after 60 h. It is hypothesized that the early formation of H_2_O on the cathode side, which deposited on the active sites of the LSCF cathode, hindered the activation process of O^2−^. This led to an increasing polarization resistance. However, after 60 h, the H_2_O generated on the cathode side and the evaporative loss of H_2_O reached a stable equilibrium, resulting in a gradual stabilization of the polarization resistance. Considering the relatively thick thickness of the applied BCZY-0.5 electrolyte membrane (30 μm), further reducing the thickness of the electrolyte membrane and optimizing the cell structure may potentially improve the overall performance of the BCZY-0.5 electrolyte, providing a solid foundation for exploring low-temperature PCFCs in the future.

## 4. Conclusions

The high Zr content in BaCe_0.55_Zr_0.35_Y_0.1_O_3-δ_ necessitates sintering at a high temperature of 1400 °C to achieve a pure-phase BCZY perovskite structure. Due to the relatively poor sintering activity of BCZY, the proton transport process may encounter more grain boundaries, and the presence of pores can severely impede proton transport, significantly impacting the cell’s performance. NiO serves as an effective sintering additive that can greatly enhance the sintering activity of BCZY. The addition of a small amount of 0.5 wt.% NiO as a sintering aid for the BCZY electrolyte not only improves its sintering activity and reduces the sintering temperature but also significantly enhances the cell’s electrochemical performance. Excessive addition of NiO may introduce electronic conductivity, resulting in a decrease in open-circuit voltage and maximum power density. Moreover, when the amount of NiO added is excessive, some impurities formed by the reaction of NiO with BCZY may be pinned at grain boundaries, acting as non-proton-conductive impurities and increasing the ohmic resistance. A single cell employing BCZY-0.5 as the electrolyte exhibits a power density approaching 700 mW cm^−2^ at 700 °C, with an ohmic resistance of merely 0.189 Ω cm^2^. The stable performance of the BCZY-0.5 electrolyte over 100 h underscores its relatively excellent proton conductivity and stability. BCZY-0.5 offers significant advantages, including a lower densification temperature, enhanced stability, and reduced operating temperature, positioning it as an exceptional proton-conducting electrolyte material for low-temperature PCFC applications.

## Figures and Tables

**Table 1:** Refinement lattice parameters of BCZY prepared at 1350 °C for 5 h with varying amounts of NiO.

Samples	Space Group	a (Å)	V (Å^3^)
BCZY-0	Pm-3m	4.3736	83.66
BCZY-0.5	Pm-3m	4.3692	83.41
BCZY-1.0	Pm-3m	4.3611	82.95
BCZY-1.5	Pm-3m	4.3576	82.74
BCZY-2.0	Pm-3m	4.3531	82.49

## Data Availability

The data presented in this study are available on request from the corresponding author. The data are not publicly available due to ongoing researches using a part of the data.

## Supplementary Materials

The following supporting information can be downloaded at: https://www.mdpi.com/article/10.3390/membranes14030061/s1, Table S1. Comparison of conductivity of BCZY, BCZY-0.5, BCZY-1.0 in air and H2 at 600–700 ℃. Figure S1. The single cell I-V and I-P diagrams of NiO-BCZY/BCZY-x (x = 0, 0.5, 1.0, 1.5, 2.0)/BCZY-LSCF with a cell structure tested in 700 ℃, 650 ℃ and 600 ℃. Figure S2. EIS diagrams of NiO-BCZY/BCZY-x (x = 0, 0.5, 1.0, 1.5, 2.0)/BCZY-LSCF with a cell structure tested in 700 ℃, 650 ℃ and 600 ℃. Figure S3. SEM-EDS mapping results for the surface of the BCZY-0.5 electrolyte membrane after testing (a–h).

## References

[1] Ni M., Shao Z. (2020). Fuel cells that operate at 300° to 500 °C. Science.

[2] Cao J., Ji Y., Shao Z. (2022). Perovskites for protonic ceramic fuel cells: A review. Energy Environ. Sci..

[3] Yang L., Wang S., Blinn K., Liu M., Liu Z., Cheng Z., Liu M. (2009). Enhanced sulfur and coking tolerance of a mixed ion conductor for SOFCs: BaZr_0.1_Ce_0.7_Y_0.2-*x*_Yb_*x*_O_3-delta_. Science.

[4] Choi S., Kucharczyk C.J., Liang Y., Zhang X., Takeuchi I., Ji H.-I., Haile S.M. (2018). Exceptional power density and stability at intermediate temperatures in protonic ceramic fuel cells. Nat. Energy.

[5] Papac M., Stevanović V., Zakutayev A., O’Hayre R. (2020). Triple ionic–electronic conducting oxides for next-generation electrochemical devices. Nat. Mater..

[6] Bai H., Leng Z., Chen T., Zhang B., Chu J., Zhang Y., Zhou Q., Zhou J., Wang S. (2023). Synthesis and characterization of PrBa_0.5_Sr_0·5_Co_2-x_Fe_x_O_5+δ_ (x = 0.5, 1.0, 1.5, 2.0) as oxygen electrode for proton-conducting solid oxide cells. Int. J. Hydrogen Energy.

[7] Zhu L., Cadigan C., Duan C., Huang J., Bian L., Le L., Hernandez C.H., Avance V., O’Hayre R., Sullivan N.P. (2021). Ammonia-fed reversible protonic ceramic fuel cells with Ru-based catalyst. Commun. Chem..

[8] Duan C., Kee R., Zhu H., Sullivan N., Zhu L., Bian L., Jennings D., O’Hayre R. (2019). Highly efficient reversible protonic ceramic electrochemical cells for power generation and fuel production. Nat. Energy.

[9] Yatoo M.A., Habib F., Malik A.H., Qazi M.J., Ahmad S., Ganayee M.A., Ahmad Z. (2023). Solid-oxide fuel cells: A critical review of materials for cell components. MRS Commun..

[10] Nikodemski S., Tong J., Duan C., O’Hayre R. (2016). Ionic transport modification in proton conducting BaCe_0.6_Zr_0.3_Y_0.1_O_3−δ_ with transition metal oxide dopants. Solid State Ion..

[11] Tong Y., Dai M., Chen C., Zhan Z. (2022). Protonic ceramic cells with thin BaZr_0.8_Y_0.2_O_3-δ_ electrolytes for stable separation of H_2_ from H_2_–CO_2_ mixtures. Int. J. Hydrogen Energy.

[12] Fabbri E., Pergolesi D., D’Epifanio A., Di Bartolomeo E., Balestrino G., Licoccia S., Traversa E. (2008). Design and fabrication of a chemically-stable proton conductor bilayer electrolyte for intermediate temperature solid oxide fuel cells (IT-SOFCs). Energy Environ. Sci..

[13] Fop S., McCombie K.S., Wildman E.J., Skakle J.M.S., Irvine J.T.S., Connor P.A., Savaniu C., Ritter C., McLaughlin A.C. (2020). High oxide ion and proton conductivity in a disordered hexagonal perovskite. Nat. Mater..

[14] Li M., Hua B., Luo J.-l., Jiang S.P., Pu J., Chi B., Jian L. (2015). Carbon-tolerant Ni-based cermet anodes modified by proton conducting yttrium- and ytterbium-doped barium cerates for direct methane solid oxide fuel cells. J. Mater. Chem. A.

[15] Danilov N.A., Starostina I.A., Starostin G.N., Kasyanova A.V., Medvedev D.A., Shao Z. (2023). Fundamental Understanding and Applications of Protonic Y- and Yb-Coped Ba(Ce,Zr)O_3_ Perovskites: State-of-the-Art and Perspectives. Adv. Energy Mater..

[16] Xing Y., Zhu B., Hong L., Xia C., Wang B., Wu Y., Cai H., Rauf S., Huang J., Asghar M.I. (2022). Designing High Interfacial Conduction beyond Bulk via Engineering the Semiconductor–Ionic Heterostructure CeO_2−δ_/BaZr_0.8_Y_0.2_O_3_ for Superior Proton Conductive Fuel Cell and Water Electrolysis Applications. ACS Appl. Energy Mater..

[17] Sun Z., Fabbri E., Bi L., Traversa E., Koc R. (2011). Electrochemical Properties and Intermediate-Temperature Fuel Cell Performance of Dense Yttrium-Doped Barium Zirconate with Calcium Addition. J. Am. Ceram. Soc..

[18] Zhao Z., Tang S., Liu X., Wang K., Yang T., Cheng M., Shao Z. (2023). Preparation, characterization and application of BaZr0.1Ce0.7Y0.2O3-δ for a high-performance and stable proton ceramic electrochemical cell. Int. J. Hydrogen Energy.

[19] An H., Lee H.-W., Kim B.-K., Son J.-W., Yoon K.J., Kim H., Shin D., Ji H.-I., Lee J.-H. (2018). A 5 × 5 cm^2^ protonic ceramic fuel cell with a power density of 1.3 W cm–2 at 600 °C. Nat. Energy.

[20] Choudhary B., Besra L., Anwar S., Anwar S. (2023). La_2_Ce_2_O_7_ based materials for next generation proton conducting solid oxide cells: Progress, opportunity and future prospects. Int. J. Hydrogen Energy.

[21] Wang B., Bi L., Zhao X.S. (2018). Exploring the role of NiO as a sintering aid in BaZr_0.1_Ce_0.7_Y_0.2_O_3-δ_ electrolyte for proton-conducting solid oxide fuel cells. J. Power Sources.

[22] Wachsmanand E.D., Lee K.T. (2011). Lowering the Temperature of Solid Oxide Fuel Cells. Science.

[23] Choi M., Paik J., Kim D., Woo D., Lee J., Kim S.J., Lee J., Lee W. (2021). Exceptionally high performance of protonic ceramic fuel cells with stoichiometric electrolytes. Energy Environ. Sci..

[24] Li Y., Guo R., Wang C., Liu Y., Shao Z., An J., Liu C. (2013). Stable and easily sintered BaCe_0.5_Zr_0.3_Y_0.2_O_3−δ_ electrolytes using ZnO and Na2CO3 additives for protonic oxide fuel cells. Electrochim. Acta.

[25] Kim H.-W., Seo J., Yu J.H., Yun K.S., Joo J.H., Moon J., Park H.J. (2021). Effect of cerium on yttrium-doped barium zirconate with a ZnO sintering aid: Grain and grain boundary protonic conduction. Ceram. Int..

[26] Babilo P., Haile S.M. (2005). Enhanced Sintering of Yttrium-Doped Barium Zirconate by Addition of ZnO. J. Am. Ceram. Soc..

[27] Ricote S., Bonanos N. (2010). Enhanced sintering and conductivity study of cobalt or nickel doped solid solution of barium cerate and zirconate. Solid State Ion..

[28] Tan X., Shen Z., Bokhari A., Ali W., Han N. (2023). Effect of Co_2_O_3_ as sintering aid on perovskite BaCe_0.8_Y_0.2_O_3-δ_ proton conductive membrane for hydrogen separation. Int. J. Hydrogen Energy.

[29] Liu Z., Wang X., Liu M., Liu J. (2018). Enhancing sinterability and electrochemical properties of Ba(Zr_0.1_Ce_0.7_Y_0.2_)O_3-δ_ proton conducting electrolyte for solid oxide fuel cells by addition of NiO. Int. J. Hydrogen Energy.

[30] Li Z., He Q., Wang C., Xu Q., Guo M., Bello I.T., Ni M. (2022). Ethylene and power cogeneration from proton ceramic fuel cells (PCFC): A thermo-electrochemical modelling study. J. Power Sources.

[31] Zvonareva I., Fu X.-Z., Medvedev D., Shao Z. (2022). Electrochemistry and energy conversion features of protonic ceramic cells with mixed ionic-electronic electrolytes. Energy Environ. Sci..

[32] Shin Y., Kim Y.-d., Sanders M., Harvey S.P., Walker M., O’Hayre R. (2022). Tuning the Co/Fe ratio in BaCo_x_Fe_0.8−x_Zr_0.1_Y_0.1_O_3−δ_, a promising triple ionic and electronic conducting oxide, to boost electrolysis and fuel cell performance. J. Mater. Chem. A.

[33] Qu G., Akbar M., Jin B., Yang W., Wang X., Dong W., Afzal M., Wang H., Xia C. (2023). Enhancing the Performance of the p-n Heterostructure Electrolyte for Solid Oxide Fuel Cells via A-Site-Deficiency Engineering. ACS Appl. Mater. Interfaces.

[34] Bian W., Wu W., Wang B., Tang W., Zhou M., Jin C., Ding H., Fan W., Dong Y., Li J. (2022). Revitalizing interface in protonic ceramic cells by acid etch. Nature.

[35] Lee M., Gan Y., Yang C., Ren C., Xue X. (2022). Fabrication and accelerated long-term stability test of asymmetrical hollow fiber-supported thin film oxygen separation membrane. J. Membr. Sci..

[36] Tong J., Clark D., Bernau L., Subramaniyan A., O’Hayre R. (2010). Proton-conducting yttrium-doped barium cerate ceramics synthesized by a cost-effective solid-state reactive sintering method. Solid State Ion..

[37] Jing J., Pang J., Chen L., Zhang H., Lei Z., Yang Z. (2022). Structure, synthesis, properties and solid oxide electrolysis cells application of Ba(Ce, Zr)O3 based proton conducting materials. Chem. Eng. J..

[38] Chen M., Chen D., Wang K., Xu Q. (2019). Densification and electrical conducting behavior of BaZr_0.9_Y_0.1_O_3-δ_ proton conducting ceramics with NiO additive. J. Alloys Compd..

[39] Wang Z., Ding L., Yu S., Xu H., Hao X., Sun Y., He T. (2022). Effect of Two Different ZnO Addition Strategies on the Sinterability and Conductivity of the BaZr_0.4_Ce_0.4_Y_0.2_O_3−δ_ Proton-Conducting Ceramic Electrolyte. ACS Appl. Energy Mater..

[40] Guo R., Li D., Guan R., Kong D., Cui Z., Zhou Z., He T. (2022). Sn–Dy–Cu Triply Doped BaZr_0.1_Ce_0.7_Y_0.2_O_3−δ_: A Chemically Stable and Highly Proton-Conductive Electrolyte for Low-Temperature Solid Oxide Fuel Cells. ACS Sustain. Chem. Eng..

[41] Rui G., Lei Y., Yaowen W., Youcheng X., Rui G., Mengjiao W., Tianmin H. (2022). Enhanced sintering and electrical properties of proton-conducting electrolytes through Cu doping in BaZr_0.5_Ce_0.3_Y_0.2_O_3-δ_. Ceram. Int..

[42] Sun W., Shi Z., Liu M., Bi L., Liu W. (2014). An Easily Sintered, Chemically Stable, Barium Zirconate-Based Proton Conductor for High-Performance Proton-Conducting Solid Oxide Fuel Cells. Adv. Funct. Mater..

[43] Chen M., Zhou M., Liu Z., Liu J. (2022). A comparative investigation on protonic ceramic fuel cell electrolytes BaZr_0.8_Y_0.2_O_3-δ_ and BaZr_0.1_Ce_0.7_Y_0.2_O_3-δ_ with NiO as sintering aid. Ceram. Int..

[44] Wan Y., He B., Wang R., Ling Y., Zhao L. (2017). Effect of Co doping on sinterability and protonic conductivity of BaZr_0.1_Ce_0.7_Y_0.1_Yb_0.1_O_3−δ_ for protonic ceramic fuel cells. J. Power Sources.

[45] Han Z., Dong H., Yang Y., Yang Z. (2022). Achieving Robust Redox Stability of SOFC through Ni-YSZ Anode Layer Thinning and Inert Support Mechanical Compensation. ACS Appl. Energy Mater..

[46] Zhong F., Han C., Luo Y., Zhou C., Chen C., Lin L., Cai G., Jiang L. (2021). Site-oriented design of spinel Mg_x_NiMn_2-x_O_4-δ_ as cathode material of intermediate-temperature direct ammonia solid oxide fuel cell. J. Power Sources.

[47] Wang W., Zhang X., Khan K., Wu H., Zhang D., Yang Y., Jiang Y., Lin B. (2021). Enhanced ORR activity of A-site deficiency engineered BaCo_0·4_Fe_0·4_Zr_0·1_Y_0·1_O_3-δ_ cathode in practical YSZ fuel cells. Int. J. Hydrogen Energy.

[48] Wang M., Ma T., Wang H., Yu S., Bi L. (2023). Microwave sintering coupled with sintering aids for proton-conducting oxide membranes. Ceram. Int..

[49] Zhou C., Sunarso J., Song Y., Dai J., Zhang J., Gu B., Zhou W., Shao Z. (2019). New reduced-temperature ceramic fuel cells with dual-ion conducting electrolyte and triple-conducting double perovskite cathode. J. Mater. Chem. A.

