# Supplementary material for: Effect of NiO Addition on the Sintering and Electrochemical Properties of BaCe0.55Zr0.35Y0.1O3-δ Proton-Conducting Ceramic Electrolyte
Source: Membranes (Basel). 2024 Feb 27;14(3):61. doi: 10.3390/membranes14030061 (PMC10972099; doi:10.3390/membranes14030061)
Supplement: Supplementary file 1 [file membranes-14-00061-s001.zip › membranes-2860473-supplementary.pdf]

Supporting Information

Effect of NiO Addition on the Sintering and Electrochemical Properties of $BaCe_{0.55}Zr_{0.35}Y_{0.1}O_{3-\delta}$ Proton-Conducting Ceramic Electrolyte

Chengxin Peng[a,†], Bingxiang Zhao[a,b,†], Xie Meng[b], Xiaofeng Ye[b,c], Ting Luo[b], Xianshuang Xin[b,c], Zhaoyin Wen[b,c*]*

[a] *School of Materials and Chemistry, University of Shanghai for Science and Technology, Shanghai 200093, P. R. China*

[b] *The State Key Laboratory of High Performance Ceramics and Superfine Microstructure Shanghai Institute of Ceramics, Chinese Academy of Sciences, Shanghai 200050, P. R. China*

[c] *Center of Materials Science and Optoelectronics Engineering, University of Chinese Academy of Sciences, Beijing 100049, P. R. China*

* Corresponding authors: E-mail: xinxianshuang@mail.sic.ac.cn, zywen@mail.sic.ac.cn

Table S1. Comparison of conductivity of BCZY, BCZY-0.5, BCZY-1.0 in air and H_2 at 600-700℃.

σ (mS cm^{-1})	BCZY-0		BCZY-0.5		BCZY-1.0	
	Air	H_2	Air	H_2	Air	H_2
600℃	1.5	2.3	6.7	8.4	6.6	7.7
650℃	2.2	3.0	9.5	10.0	8.8	9.3
700℃	3.0	3.7	12.2	11.3	11.3	10.8

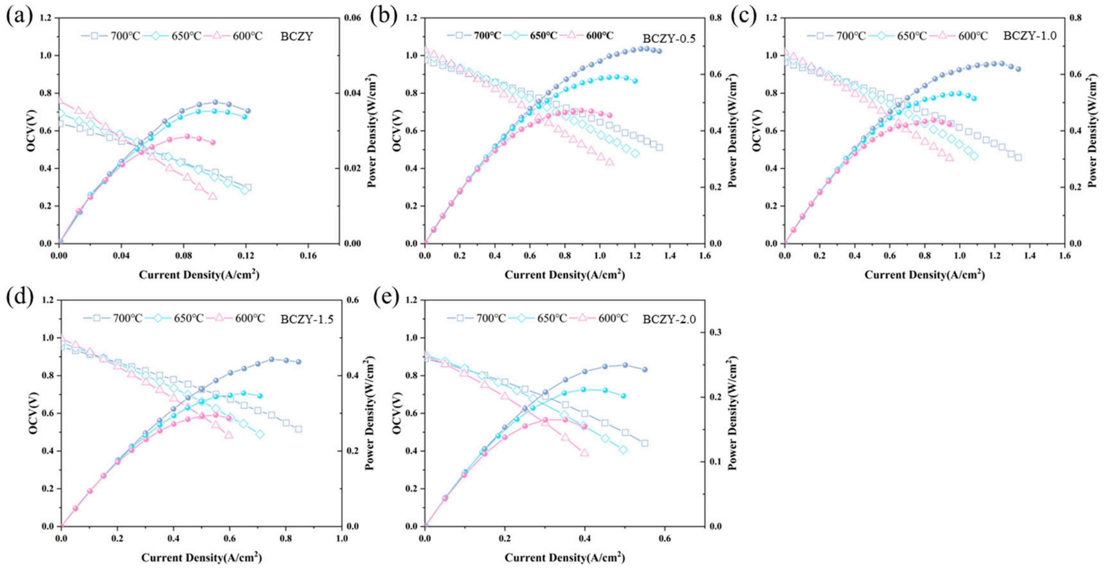

Figure S1. The single cell I-V and I-P diagrams of NiO-BCZY/BCZY-x (x=0, 0.5, 1.0, 1.5, 2.0)/BCZY-LSCF with a cell structure tested in 700℃、650℃ and 600℃.

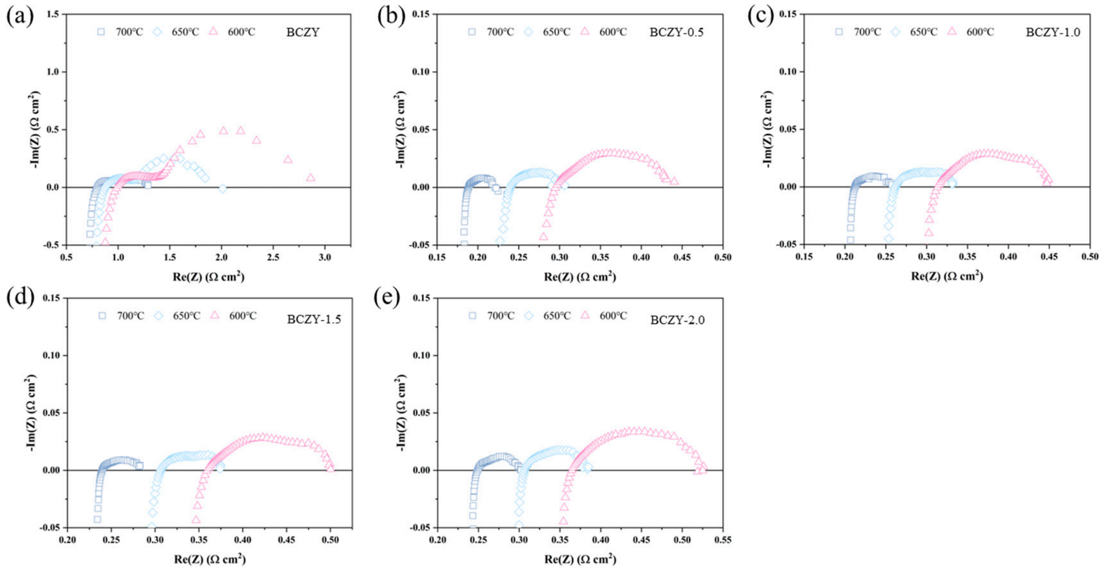

Figure S2. EIS diagrams of NiO-BCZY/BCZY-x (x=0, 0.5, 1.0, 1.5, 2.0)/BCZY-LSCF with a cell structure tested in 700℃、650℃ and 600℃.

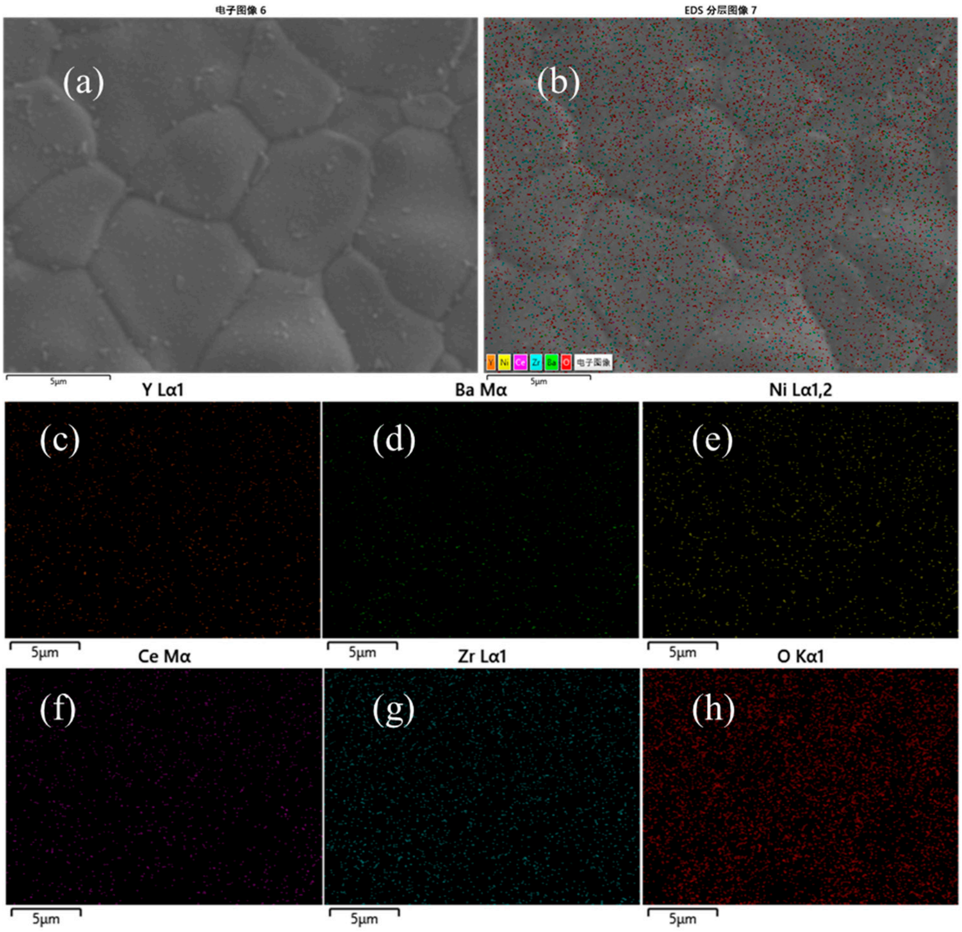

Figure S3. SEM-EDS mapping results for the surface of the BCZY-0.5 electrolyte membrane after testing (a)-(h).
